# Cell Blebbing in Confined Microfluidic Environments

**DOI:** 10.1371/journal.pone.0163866

**Published:** 2016-10-05

**Authors:** Markela Ibo, Vasudha Srivastava, Douglas N. Robinson, Zachary R. Gagnon

**Affiliations:** 1 Johns Hopkins University, Department of Chemical and Biomolecular Engineering, Baltimore, MD, 21218, United States of America; 2 Johns Hopkins University School of Medicine, Department of Cell Biology, Baltimore, MD, 21205, United States of America; Université de Genève, SWITZERLAND

## Abstract

Migrating cells can extend their leading edge by forming myosin-driven blebs and F-actin-driven pseudopods. When coerced to migrate in resistive environments, *Dictyostelium* cells switch from using predominately pseudopods to blebs. Bleb formation has been shown to be chemotactic and can be influenced by the direction of the chemotactic gradient. In this study, we determine the blebbing responses of developed cells of *Dictyostelium discoideum* to cAMP gradients of varying steepness produced in microfluidic channels with different confining heights, ranging between 1.7 μm and 3.8 μm. We show that microfluidic confinement height, gradient steepness, buffer osmolarity and Myosin II activity are important factors in determining whether cells migrate with blebs or with pseudopods. *Dictyostelium* cells were observed migrating within the confines of microfluidic gradient channels. When the cAMP gradient steepness is increased from 0.7 nM/μm to 20 nM/μm, cells switch from moving with a mixture of blebs and pseudopods to moving only using blebs when chemotaxing in channels with confinement heights less than 2.4 μm. Furthermore, the size of the blebs increases with gradient steepness and correlates with increases in myosin-II localization at the cell cortex. Reduction of intracellular pressure by high osmolarity buffer or inhibition of myosin-II by blebbistatin leads to a decrease in bleb formation and bleb size. Together, our data reveal that the protrusion type formed by migrating cells can be influenced by the channel height and the steepness of the cAMP gradient, and suggests that a combination of confinement-induced myosin-II localization and cAMP-regulated cortical contraction leads to increased intracellular fluid pressure and bleb formation.

## Introduction

During migration, motile cells must restrict protrusive activity to their periphery if they are to migrate efficiently, and during chemotaxis, these projections must be controlled by the chemotactic gradient. Migrating cells move by extending their leading edge using two main types of protrusions: pseudopods (or lamellipods) driven by actin polymerization, and from pressure-driven membrane blebs [1,2]. Blebs are rapidly expanding rounded membrane protrusions that form when the cell membrane separates from the cortex. They grow as a result of intracellular pressure created by myosin II-mediated cortical contraction [3–5]. Blebbing occurs during cytokinesis [6], cell spreading [7] and apoptosis [8]; however, recent work demonstrates that blebs also play a role as leading edge protrusions in restrictive three-dimensional environments [9–15].

*Dictyostelium* amoebae can also move using blebs [16–19]. *Dictyostelium discoideum* is a fast-moving genetically accessible single cell organism, and has become an ideal model for studying basic aspects of cell motility [20,21]. When starved, *Dictyostelium* cells undergo a developmental process where signaling proteins are upregulated, and after a few hours, they develop a polarized morphology as well as the ability to sense and chemotax towards sources of cyclic adenosine 3’,5’-monophosphate (cAMP). Oscillatory pulses of cAMP coordinate and recruit chemotaxing cells to form multicellular structures and these cells make a natural transition from moving individually on a planar surface to moving within confined three-dimensional aggregates [22].

During chemotaxis under buffer, *Dictyostelium* move mainly using F-actin-driven pseudopods, but switch to using blebs when migrating through mechanically resistant environments [17]. This behavior is usually observed using an elastic overlay, such as agarose, where cells are coerced to migrate underneath and deform the overlay to continue towards a nearby well containing cAMP. Cells passing under the agarose exert mechanical force on the overlay and in doing so experience mechanical resistance from it. The degree of mechanical resistance can be controlled using different agarose concentrations, and work has shown that when the stiffness of the agarose is increased, cell blebbing increases [17].

Chemotactic gradients can also control the position where blebs preferentially form [17]. During chemotaxis, PI3-kinase accumulates at the leading edge of migrating *Dictyostelium* cells [23,24]. *Dictyostelium* cell blebbing is also strongly polarized up-gradient and is regulated through PI3-kinase [17]. In *PI3-kinase* null cells, where all five “type-1” PI3-kinases in the genome have been knocked out, cells migrate using significantly less blebs than parental cells [17]. Previous work also shows that the chemotactic response of *Dictyostelium* cells is dependent on gradient steepness [25].

Blebbing requires sufficient intracellular fluid pressure to drive membrane expansion [2–4]. This blebbing is mediated through myosin II-induced contraction of the cortex, where both heavy and light chain mutants are unable to bleb under buffer or agarose [17,26–28]. Myosin II activity in *Dictyostelium* is stimulated by cAMP and regulated, in part, through phosphorylation of its regulatory light chain, which is simulated by cAMP signaling through downstream guanylyl-cyclases and cyclic-GMP-binding proteins. Chemotactic stimulation of *Dictyostelium* cells therefore results in a transient increase in cGMP and phosphorylation of myosin II heavy and regulatory light chains [29]. Because cAMP controls where blebs form, mediates myosin II contraction, and influences *Dictyostelium* chemotactic motility, we sought to investigate the influence of cAMP steepness and myosin II activity on cell blebbing during cell migration in confined environments.

A major disadvantage of utilizing agarose overlays for observing migrating cell populations is that they do not incorporate well-controlled chemical and mechanical constraints. During migration, cells must squeeze and move underneath the agarose. During this process they locally detach the overlay and move it upward. Cells therefore face spatial variations in height and mechanical resistance since the cell must deform and lift the agarose off the substrate surface to move forward. During this process, the cell’s leading edge is more confined. The chemical gradient is also difficult to control since is not stable and continues to change over time [25]. Furthermore, under agarose environments do not allow for the removal of any chemicals released by the cells, which can influence their environment and the cAMP gradient. As an alternative to agarose, we studied the influence of cell confinement and gradient steepness on blebbing in a microfluidic environment. Unlike agarose overlays, cells can be controllably coerced into precisely controlled microchannels containing stable linear chemical gradients. The degree of mechanical resistance can be varied by simply changing the height of the microchannels that cells migrate through [30]. So far, under agarose assays have shown that *Dictyostelium* cells use blebs to migrate in resistive confined environments. However, the influence of gradient steepness on blebbing has not been investigated. Microfluidics has been used to produce stable gradients for *in vitro* cell migration studies. Moreover, many different types of gradient geometries have been used to study migrating cell populations both on 2D and 3D substrates [31–39]. Microfluidic gradients have been used to examine the migration of tumor cells [38,40–42], neutrophils [43], and the localization of internal molecules at high resolution in *Dictyostelium* [44]. However, these devices have not been extended to analyzing the influence of chemical gradient steepness on cell protrusion type. Because bleb-driven movement is chemotactic [18] and triggered by applied mechanical resistance, this raises further questions about how cAMP gradient steepness influences bleb formation and how this influences the balance between actin polymerization and myosin contractility to produce blebs.

In this work, we offer insight into the role that confined environments can play in promoting the formation of blebs during chemotaxis. In particular, we make use of microfluidics to create confining microchannels with stable linear cAMP gradients. *Dictyostelium* cells are loaded into non-confined microchannel chambers and coerced to migrate through confined channels with a fixed width (50 μm) and varying height (1.7, 2.4, or 3.8 μm). For each confinement experiment, we quantified the rate to which cells formed blebs or pseudopods at different cAMP gradient steepness, ranging between 0.7–20 nM/μm. We observed that cells migrated through all channels, but the degree of confinement influenced the degree to which cells moved with blebs as is consistent with other reports conducted using agarose overlays. Blebbing was observed to increase with increasing steepness of the cAMP gradient, and cells produced larger blebs when the gradient was increased. Moreover, when the internal cell pressure was reduced using buffer containing sorbitol, cells formed smaller blebs with reduced frequency. Myosin-II concentration at the cortex was seen to be independent of gradient steepness, but increased by as much as 250% when cells were coerced to migrate in channels 1.7 μm in height. Finally, inhibition of myosin-II by blebbistatin or through the use of Myosin II-null cells led to a reduction in both the percentage and size of blebs used by migrating confined cells.

## Materials and Methods

### Mutant Strains and Cell Development

Cells were derived from the axenic strain Ax2 of *D*. *discoideum*. Cells were grown in 1.5X HL-5 medium with glucose (FORMEDIUM Ltd.)(1L H2O, 15 g proteose peptone, 3.9 g bacto-yeast extract, 3.0 g glucose, 0.13 g Na_2_HPO_4_-7H_2_O, 0.13 g KH_2_PO_4_), and all experiments were performed at 22°C. Ax2 was transformed with markers for F-actin (GFP-LimEΔcoil), the plasma membrane (cAR1-mCherry, kindly provided by P.N Devreotes) or myosin II (GFP-myosin II). Transformed cell lines were selected with G418 and Hygromyocin B. When exponentially growing cells reached a concentration of 2–4 x 10^6^ cells/mL, they were washed free of growth medium in development buffer (DB; 5 mM KHPO_4_, 5 mM Na_2_HPO_4_, 1 mM CaCl_2_, 2 mM MgCl_2_ pH 6.4) and resuspended in DB buffer at a concentration of 2 x 10^7^ cells/mL. Cells were then starved by shaking at 180 rpm for 4 h with pulses of 100 nM cAMP added every 6 min after the first hour.

### Microfluidic Gradient Generator

All microfluidic devices were made of PDMS (Momentive, RTV 615A) using standard soft lithographic methods. Briefly, a 1:10 mixture of PDMS elastomer and curing agent was poured atop a lithographically fabricated polymer mold, cured, and gently peeled off. Fluid ports were punched into the PDMS using a 0.75 mm biopsy punch (Ted Pella, Inc.), the microfluidic device and coverslip were exposed to oxygen plasma (Jelight, Model 42A) and immediately aligned and sealed under an inverted microscope, and the assembled device was baked at 90°C for 2 hours to ensure strong bonding. Buffer solutions (DB and DB + cAMP) were delivered to each microfluidic inlet at a flow rate of 1.5 μL/min using a low-cost external pressure driven flow controller [45]. A solution of Alexa Fluor 647 hydrazide (Invitrogen) was dissolved in the cAMP buffer to characterize the concentration profile of cAMP because of their comparable molecular weight.

**Fig 1:**
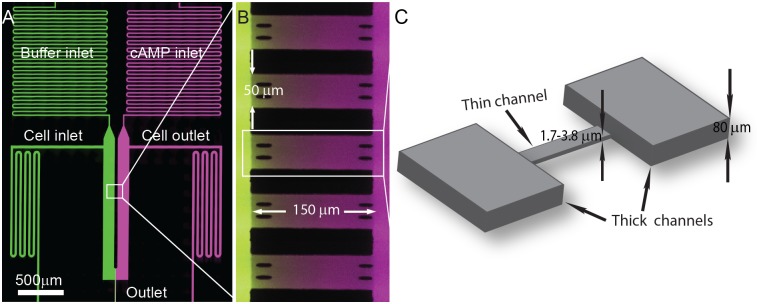
Microfluidic device for studying cell blebbing in confinement. (**A**) Confocal micrograph of the microfluidic gradient generator. The device consists of an array of microchannel gradient channels aligned perpendicular to main flow channels. Buffer is introduced through “Buffer inlet” (shown in green), and buffer with cAMP is introduced through “cAMP inlet” (shown in purple). Cells are loaded into the device by flowing a cell suspension into the “Cell inlet”. (**B**) Linear concentration profiles in gradient channels are established through molecular diffusion between the two main flow channels. Each gradient channel has a length of 150 μm and a width of 50 μm. (**C**) The “thin” confinement channels are between 1.7 and 3.8 μm in height connected to the main “thick” flow channels that are 80 μm in height.

To create regions of controlled microfluidic cell confinement, we fabricated “thin” gradient channels orthogonal to “thick” buffer channels using a two-step soft-lithography process inspired from previous chemotaxis work conducted by [44]. First, the “thin” confinement channels were patterned on a silicon wafer using a low viscosity photoresist. A second “thick” photoresist was spin-coated onto the wafer and patterned to create non-confining gradient side-channels and cell-seeding chambers. Once the microchannel mold was completed, PDMS elastomer was poured atop and cured. The final cured PDMS slab was peeled off the wafer and bonded to a glass slide using a brief oxygen plasma treatment to form the completed gradient device. The final device consisted of confining gradient chambers orthogonally-aligned with two larger main channels. These channels were designed with a length L = 150 μm and width W = 50 μm, with various microchannel heights H ranging from 1.7 to 3.8 μm (Fig 1a). To prevent shallow channels from collapsing during fabrication, we installed small pillars at each confined microchannel entrance and exit. The pillars or the PDMS did not impede cell invasion or impact the formation of the passive gradient as *Dictyostelium* cells can form actin foci on both surfaces, glass and PDMS [44] and their adhesion is not affected by surface hydrophobicity [46].

Two inlets upstream of the gradient chambers served as loading ports for buffer and chemoattractant, while the two downstream inlets were used for cell seeding into the device. The gradient generator operated using constant pressure-driven flow. The upstream inlets were pressurized at equal pressures using an external constant pressure source to deliver cell buffer (0 μM cAMP) to one side of the device and chemoattractant to the other side. The concentration of cAMP solutions used to generate the gradients were 0.1 μM cAMP, 0.5 μM cAMP, 2 μM cAMP, and 3 μM cAMP. Because the microfluidic design is symmetric and the fluid pressures are equal, no fluid flow is produced across the two main flow channels and stable gradients of chemoattractant are generated within the confinement chambers through molecular diffusion between these two flow channels (S1 Fig). To visualize the diffusion-driven gradient (Fig 1b), fluorescent Alexa Fluor 488 and Alexa Fluor 647 dyes were introduced into the buffer and chemoattractant streams, respectively. The gradient was rapidly established across the confinement slots and maintained indefinitely due to the continuous passive molecular diffusion between the two flowing buffer chemoattractant reservoir streams (Fig 1c).

### Live Cell Imaging

Measurements were performed with a Nikon Eclipse Ti swept field confocal microscope (70 μm confocal slit, Nikon/Prairie Technologies) equipped with an Andor iXon 897 camera, four 50 mW solid-state lasers for excitation, and a 60x oil objective (NA 1.49), which produced a 1.1 μm-thick optical slice. Protrusion activity was verified using brightfield microscopy to ensure that the thickness of the confocal optical slice was adequate to capture all blebs across the cell cross section. Cell imaging was performed in DB buffer and cells were seeded into the microfluidic chamber at a concentration of 2.5 x 10^5^ cells/mL. Cell activity was captured in 4 minute increments. Cell speed was determined by dividing total distance of the cell trajectory by 4 min and the chemotactic velocity was calculated by dividing the net distance cells travel towards the cAMP source by 4 min. The chemotaxis index was obtained by dividing the net distance towards the source by the total migration distance. These calculations were performed using ImageJ (Fiji) with the MtrackJ plugin. Cell trajectories were calculated using the ImageJ chemotaxis plugin. Lastly, the bleb surface area was calculated based on measurments obtained with two dimensional confocal micrographs; we measured the perimeter of each cell bleb and converted this data into a 2D surface area. Results of blebbing for different microchannel heights were compared using one way ANOVA and unpaired Student’s *t*-tests. Differences were considered significant at P < 0.05. Each experiment was performed with between 20–50 cells from several microfluidics gradient channels.

## Results and Discussion

### Bleb-Driven Motility under Microfluidic Confinement

Blebbing was previously observed in *Dictyostelium* cells as they migrated under agarose towards a nearby well containing cAMP [17]. The proportion of blebs compared with total cellular projections increased from 20% under buffer and approached upwards of 100% in cells moving under overlays with more than 1% agarose. As might be expected, cells were observed to flatten when moving under the agarose, with the cell height decreasing with increasing agarose stiffness. When non-confined under buffer, for example, cells migrated with an average height of ~ 8 μm, while at an agarose concentration above 1% cells were flattened and migrated with a height of ~ 3.5 μm. Based on the confined cell height under agarose, we fabricated microfluidic confinement chambers for quantifying cell protrusion activity, and determined the equivalent microchannel height where blebbing was shown to play a significant role in motility.

To investigate the influence of microfluidic confinement on cell blebbing, we examined *Dictyostelium* cells migrating through channels of varying height. For each case, wild-type Ax2 cells were coerced to migrate on glass through a confinement channel using a stable linear gradient of cAMP. The channel height was controlled using standard soft lithography techniques, and the cAMP gradients were imaged using confocal microscopy (Fig 1a). To illustrate the microfluidic gradient, buffers were labeled with two different Alexa Fluor fluorescent dyes. Cells were loaded and seeded into the device by flowing a cell suspension through the cell inlet and outlet channels. The cAMP gradient was created using positive pressure to deliver continuous and equal flow rates through two microfluidic inlets, one (shown in green) containing development buffer (DB) and a second (purple) with a fixed concentration of cAMP in DB. Confinement channels of varying micron-scale heights were fabricated across the DB and DB/cAMP inlets. A gradient was quickly established by passive diffusion from one fluidic inlet to the other across these channels (Fig 1b). Cells were then imaged as they migrated from 80-μm “thick” buffer channels across a “thin” confinement channel 150 μm in length and into the adjacent cAMP/DB channel (Fig 1c).

**Fig 2:**
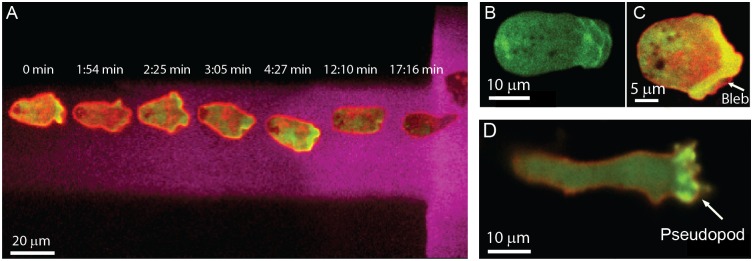
Cell migration in microfluidic confinement. (**A**) A confined *Dictyostelium* cell migrates up a cAMP gradient. The gradient is imaged using Alexa Fluor 647 hydrazide. The seven superimposed micrographs of the cell were captured (3.28 FPS) at time intervals t = 0, 1:54, 2:25, 3:05, 4:27, 12:10 and 17:16 min. (**B**) During bleb expansion F-actin scars remain behind and the newly formed bleb is almost devoid of F-actin. (**C**) When confined, blebs formed at the leading edge of a cell expressing a membrane marker (mCherry-cAR1) and F-actin reporter (GFP-LimEΔcoil). (**D**) Cells predominately formed pseudopods when migrating under buffer.

We first performed experiments to investigate the influence of microchannel height on the formation of cell blebs and observed the migration of confined *Dictyostelium* cells through a thin microfluidic channel containing a controlled cAMP gradient (Fig 2a). A solution of Alexa Fluor 647 hydrazide (Invitrogen) was dissolved in the cAMP buffer to characterize the concentration profile of cAMP because of their comparable molecular weights. We followed cell protrusion activity and formation by transforming cells with a construct that generates Green Fluorescent Protein (GFP) linked to a protein domain that specifically binds to F-actin (LimEΔcoil) (Fig 2b). The position of the cortex was simultaneously imaged relative to the cell membrane location using the cyclic AMP receptor 1 (cAR1) fused to mCherry (mCherry-cAR1) as a membrane marker (Fig 2c).

**Fig 3:**
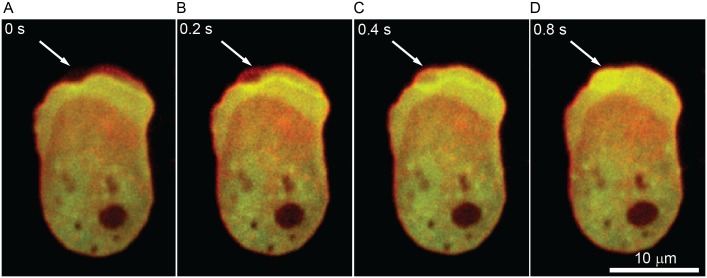
Blebs produced by a cell moving through a 1.7 μm microchannel. (**A**) A bleb forms when the cell membrane detaches from the cortex. (**B**) The bleb expands in approximately 0.2 s, leaving behind a cortical F-actin scar. (**C, D**) Initially the bleb lacks an F-actin cortex, but it is rebuilt in less than one second. Images captured at 5.12 FPS.

As cells migrated across the confinement channel, protrusions were classified in terms of the observed actin dynamics at the cell membrane. Pseudopods expand slowly and steadily, with F-actin remaining continuously associated with the membrane as they expand (Fig 2c). Blebs, however, are rapidly expanding membrane protrusions that typically appear in less than one second (Fig 3). After the membrane detaches from the cortex, blebs leave behind an F-actin “scar”, which represents the previous cortex position (Fig 3b). The F-actin scar disappears in a few seconds while at the same time new cortex builds beneath the membrane of the bleb (Fig 3c and 3d). We classified protrusions as blebs or pseudopods depending on the presence of either an actin scar or actin at the leading edge. The blebbing activity was assessed by computing the percent of blebs used to the total number of protrusions a cell used to transverse the confinement channel.

**Fig 4:**
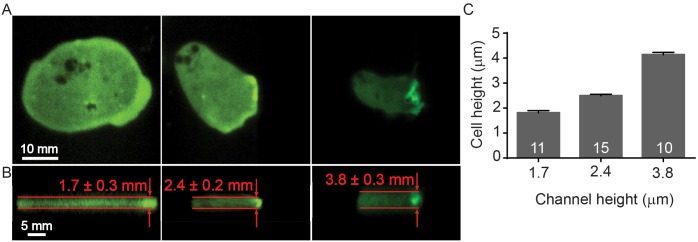
Cell confinement is controlled through microchannel height, as determined from confocal images. (**A**) Top view of cells confined using three different microchannel heights. (**B**) Side view of cells confined to heights of 1.7, 2.4 and 3.8 μm, as determined by using confocal microscopy. Fluorescence signal is from GFP-LimEΔcoil. (**C**) The measured cell height correlates with the fabricated microchannel height. Cell numbers shown on each bar. Error bars represent SEM.

*Dictyostelium* cells migrate using blebs when forced to move under agarose. Here, we extend these assays to microfluidic confinement channels for studying cell bleb formation. Previous work with agarose suggests that cells forced into a confined region ~ 3 μm in height will move predominately using blebs. Here, we investigated whether a mechanically restrictive agarose environment can be accurately translated into a microfluidic channel of fixed geometric height. Using previous agarose overlay data as reference, we examined the blebbing behavior of *Dictyostelium* cells under different microfluidic confinement heights, varying between 1.7 μm and 8 μm. Interestingly, unlike agarose where cells predominantly moved using blebs when flattened to a height of ~ 3.5 μm, we observed that cells confined to the equivalent height in a microchannel migrate using pseudopods. However, when the channel height was reduced to 2.4 μm, cells began using blebs with increased frequency. Based on these experiments, we investigated blebbing behavior using three different microchannel heights: 1.7 μm, 2.4 μm, and 3.8 μm. Each channel height was fabricated using a different microchannel mold and casting a new PDMS device. We measured the microchannel mold height (channel height) and the compared this with the height of confined migrating cells expressing GFP-LimEΔcoil using confocal microscopy (Fig 4a and 4b). The microchannel mold height matches the channel height with confined cells, indicating that the fabricated channel height was not influenced by migrating cells and cells were unable to deform the PDMS microchannel (Fig 4c). Moreover, cells were completely confined to within the given microchannel for all channel heights used in this work.

**Fig 5:**
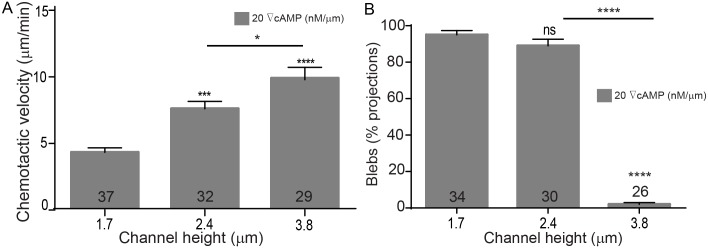
Cell migration is influenced by microfluidic confinement. *Dictyostelium* cells are observed as they migrate up a 20 nM/μm cAMP gradient. (**A**) Chemotactic cell velocity decreases as the height of the channel is reduced. (**B**) Blebbing increases as the height of the microfluidic gradient channel is reduced. Blebs given as percentage of total projections (pseudopods + blebs). Error bars represent SEM. The number of cells quantified shown on bars. **P ≤ 0.01, ***P ≤ 0.001, ****P ≤ 0.0001.

**Fig 6:**
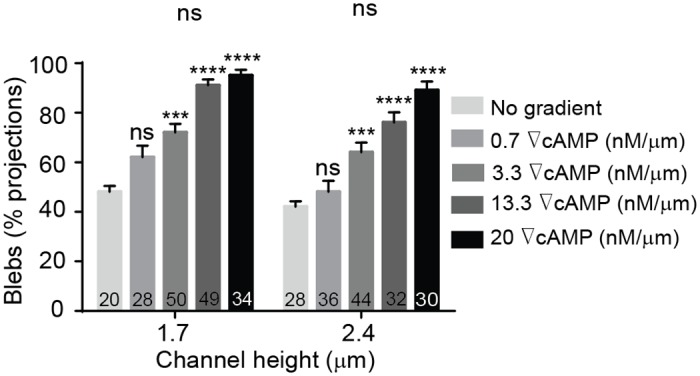
Bleb-driven movement is regulated by cAMP gradient steepness. The steepness of the cAMP gradient increases the blebbing frequency. Cell numbers are shown on bars. Error bars represent SEM. ***P ≤ 0.001, ****P ≤ 0.0001.

**Fig 7:**
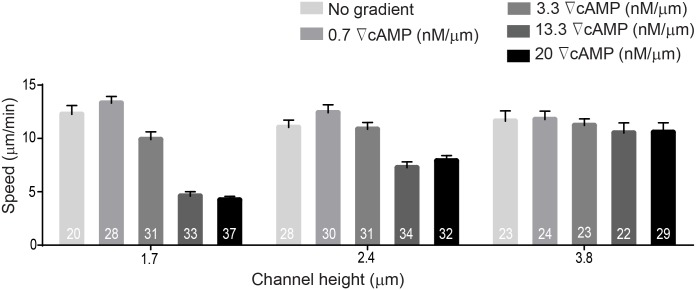
Cell speed is reduced when cells migrate largely using blebs. The Dictyostelium cell velocity is reduced with increasing the steepness of cAMP gradient at 1.7 μm and 2.4 μm. Under these conditions cells migrate using a larger percentage of blebs (see Fig 6). Cell velocity remains approximately constant when cells migrate in 3.8 μm-tall channels, were cells used very few blebs. The data shown are the mean ± SEM.

**Fig 8:**
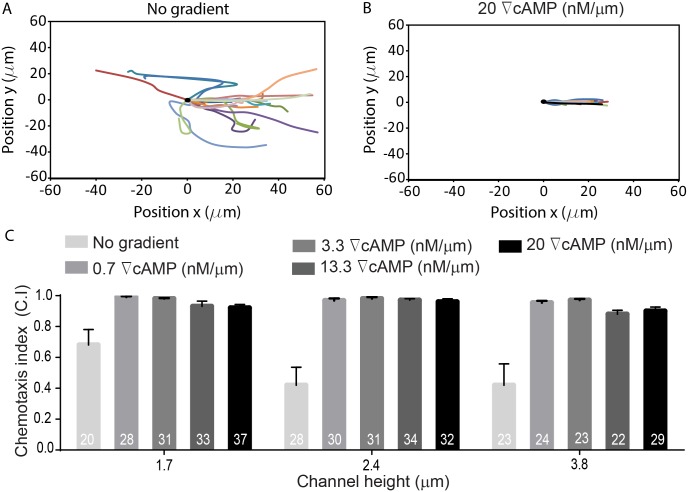
Chemotaxis index (C.I) of *Dictyostelium* is not influenced by microchannel height. The cell tracks for 20 cells are shown for cells chemotaxing in a 1.7 μm-tall channel in (**A**) no cAMP gradient and (**B**) a 20 nM/μm cAMP gradient. (**C**) The cell chemotaxis index varies from 0.9–1.0 when cells migrate in a cAMP gradient, but significantly less when no gradient is applied. The data shown are the mean ± SEM.

For each confinement experiment, developed *Dictyostelium* cells expressing mCherry-cAR1 and GFP-LimEΔcoil were seeded into a non-confining microfluidic chamber containing DB buffer. A cAMP gradient was established across the thin confinement channels and cells migrated towards the cAMP source using pseudopodia. Cells invaded the confinement channels and migrated up the cAMP gradient. During migration we quantified the chemotactic velocity (Fig 5a), the percentage of cell protrusions that were blebs (Fig 5b), the influence of the chemical gradient steepness on protrusion formation (Fig 6) and cell speed (Fig 7), and the chemotaxis index (Fig 8). The chemotactic cell velocity decreased by approximately 50% from 10 μm/min to 5 μm/min when the confinement height reduced from 3.8 μm to 1.7 μm (Fig 5a). Similar to previous bleb studies using agarose, the influence of microfluidic confinement on the cell protrusion mode was dramatic. Cells confined in 1.7 μm and 2.4 μm tall channels under a 20 nM/μm cAMP gradient predominantly migrated across the confinement channel using blebs. Cells, however, confined in a 3.8-μm channel under the identical chemical gradient steepness moved largely using pseudopodia and formed only a small number of blebs (Fig 5b).

### Influence of Gradient Steepness on Cell Blebbing

In *Dictyostelium*, gradient steepness is an important factor in determining cell directionality and chemotactic velocity. Above a threshold gradient steepness (~10^−3^ nM/μm), the chemotactic motion of *Dictyostelium* is governed by the steepness of the applied gradient and is independent of the local cAMP concentration [25,39,47]. Since cAMP gradient steepness was shown previously to impact cell movement and chemotactic velocity, we investigated whether increases in cAMP steepness impacted cell bleb formation. Because directed motion of *Dictyostelium* in linear gradients was shown previously to occur when gradient steepness was greater than 10^−3^ nM/μm [43], we used this threshold value as guidance for choosing the lowest gradient investigated in this work. Initially, we applied the gradient of 0.07 nM/μm (data not shown), however, the frequency of cells entering the channels was very low so we increased our gradient by one order of magnitude to 0.7 nM/μm. Based on this initial steepness, we used four different cAMP gradients in our microfluidic channels: 0.7 nM/μm, 3.3 nM/μm, 13.3 nM/μm, and 20 nM/μm. We then quantified the number of blebs as a percentage of total projections (blebs + pseudopods) for cells migrating under each gradient steepness in microchannels with heights of 1.7 μm and 2.4 μm (Fig 6). The influence of gradient steepness on blebbing frequency is apparent with the proportion of blebs in cells migrating confined in a 1.7-μm channel increasing from 65% at a low steepness (0.7 nM/μm) to nearly 95% for the same cells moving up a 20 nM/μm cAMP gradient. Blebbing activity was measured in the same way for a channel height of 2.4 μm where cells displayed nearly identical (statistically insignificant) behavior with gradient steepness as compared to cells confined in a 1.7-μm channel (Fig 6). Chemotactic velocity was also observed to decrease with increasing gradient steepness (Fig 7) and when the cAMP gradient was removed, cells produced significantly fewer blebs in all three channel heights and migrated with a reduced chemotactic index. The chemotactic index does not approach zero due to the biasing influence of the microchannel sidewalls; cells migrate into the wall and are directed along the channel axis. When the gradient was applied, the chemotaxis index increased and varied from 0.9 to 1 for all channel heights and cAMP gradient steepness. Therefore, cell velocity does not decrease due to a reduced bias towards cAMP, but because cells migrate using more blebs (Fig 8). This finding is consistent with previous work demonstrating that cell displacement is greater when cells migrate with pseudopods than with blebs [17]. To determine whether the blebbing was impacted by the local concentration of cAMP or by the relative gradient we quantified the number of blebs as a percentage of total number of protrusions (blebs + pseudopods) for cells migrating near the inlet and the outlet of the microfluidic gradient channels. The linear cAMP gradient provided a constant relative gradient throughout the entire length of the confinement channels, however, the local concentration of cAMP was low at the gradient inlet and at a maximum value at the high concentration outlet (S1 Fig). Results show that the percentage of blebs utilized by the cells remained the same in both low and high local cAMP concentrations (S2 Fig).

### Myosin II Activity Is influenced by Microfluidic Confinement

Our experiments illustrate that microfluidic confinement significantly influences the degree to which *Dictyostelium* cells move by using blebs. Previous work attributes increased blebbing activity to the mechanical resistance of the environment, which has been reported as “the force cells exert to deform the matrix” [17]. In our microfluidic device, however, we found that cells do not deform the microchannel during chemotaxis (Fig 4b). Therefore, cells do not experience mechanical resistance in the same way as they do under agarose since no force is necessary to deform the microfluidic channel. Confinement in microfluidic channels and migration against physical resistance under agarose, however, are related to some degree in that both environments lead to an increase in intracellular pressure. During migration, confined cells experience an increase in membrane tension when compared to non-confined movement under buffer. With this increase in membrane tension, cells require sufficient cortical tension to overcome confinement stresses and migrate. To provide structural support, we hypothesized that myosin II becomes locally associated to the actin cytoskeleton in order to increase cortical tension and provide sufficient integrity [27,48].

Myosin II activity is also associated with bleb formation in *Dictyostelium*. Heavy chain null-mutants and cells treated with blebbistatin, for example, are unable to bleb [17–19]. A key requirement for blebbing is that the cell must produce enough internal fluid pressure to drive membrane detachment and bleb expansion. Below a critical fluid pressure, blebs cannot expand [49]. Hydrostatic pressure is produced by myosin-II-mediated contraction of the actomyosin cortex. Myosin II provides multiple functions in the cell cortex, including promoting contractility, increasing cortical tension and viscoelasticity, and sensing and responding to mechanical stresses acting in the cytoskeletal network [50]. The myosin II-mediated cortical tension then combines with local surface curvature, leading to hydrostatic pressure that can promote rupture of the cortical actin network or local detachment of the plasma membrane [51]. Once the cortex is ruptured or the membrane detached, cytosol flows along the pressure gradient and forces the plasma membrane to protrude outward as an expanding bleb.

The tendency for cells to form blebs through cortical contractility suggests that cells might adapt to confinement conditions through increases in myosin activity since confined cells experience external stress from the environment and require sufficient cortical tension to overcome these forces and migrate. Interestingly, previous studies show that myosin II localizes at the cell membrane in cells undergoing chemotaxis under agarose overlay [27]. Because there is clear evidence that myosin II plays a role in controlling membrane and cortical tension [49] and producing contractile forces for bleb formation, we asked whether myosin II activity increases when cells are confined and how this correlates with blebbing activity in confinement channels of differing height and with variations in the cAMP gradient steepness.

**Fig 9:**
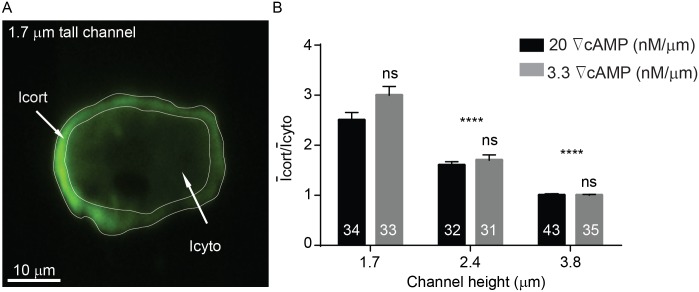
Myosin-II localizes to the cell cortex when cells are confined within microfluidic gradient channels. (**A**) Fluorescence intensity of GFP-myosin was higher at the cell cortex (Icort) than in the cytosol (Icyto). (**B**) The ratio of Icort/Icyto increased when the microfluidic confinement channel height was decreased from 3.8 μm to 1.7 μm. Cell numbers are shown on bars. Error bars represent SEM. ****P ≤ 0.0001.

**Fig 10:**
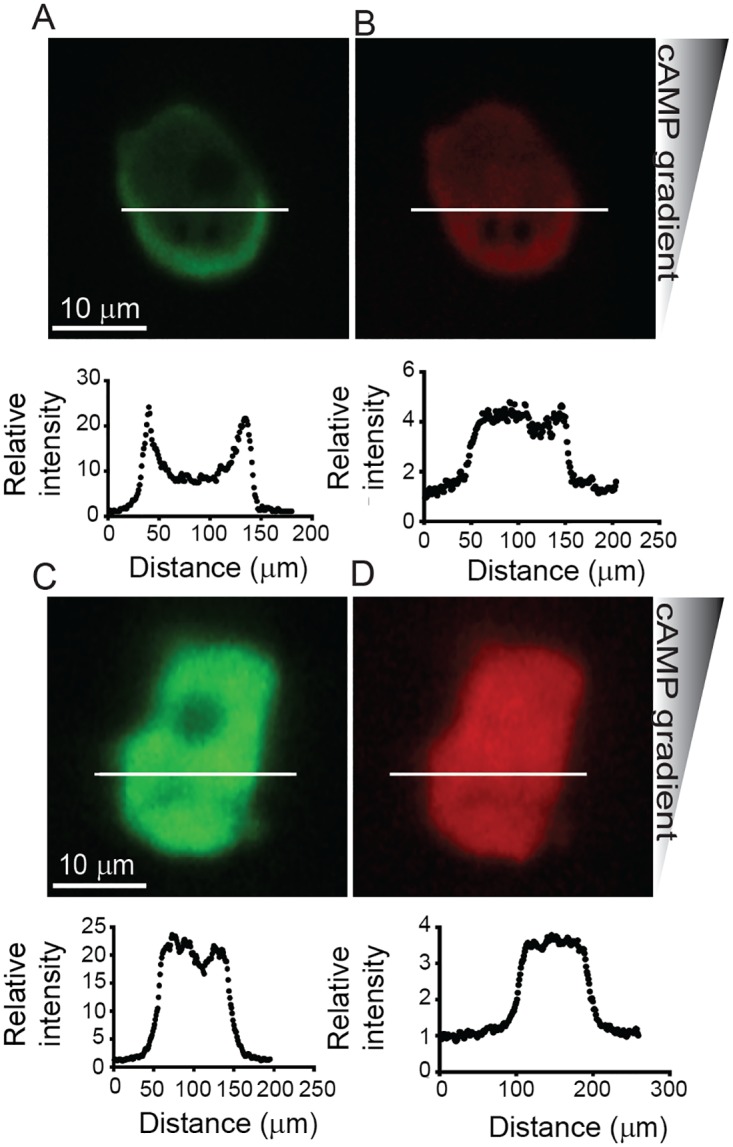
Myosin-II and cytosolic mCherry during migration under microfluidic confinement. (**A**) Myosin-II accumulates at the cortex in confined *Dictyostelium* cells, while (**B**) mCherry, used as a volume marker, does not show cortical/membrane enrichment at 1.7 μm. (**C**) Myosin-II and (**D**) mCherry do not concentrate at the cell cortex in confined cells at 3.8 μm. Cell numbers are shown on bars. Error bars represent SEM.

Cells expressing GFP-myosin II were filmed at 5–10 Hz while under different degrees of microfluidic channel confinement. Myosin II localized at the cell membrane when *Dictyostelium* was confined in a 1.7 μm channel (Fig 9a), and the degree of myosin II localization at the cortex decreased with increasing channel height (Figs 9b, 10a and 10c). Cells expressing cytosolic mCherry were also imaged while confined at 1.7 μm and 3.8 μm, and no localization was observed (Fig 10b and 10d). Our data shows that microfluidic confinement promotes increased myosin II localization at the cell cortex. However, we found that the degree of translocation is not influenced by cAMP gradient steepness (Fig 9b). GFP-myosin II was measured in cells migrating in channels with 1.7, 2.4 and 3.8 μm at two different gradients: 20 nM/μm and 3.3 nM/μm. For each channel height, myosin II activity remained unaffected by changes in gradient steepness.

**Fig 11:**
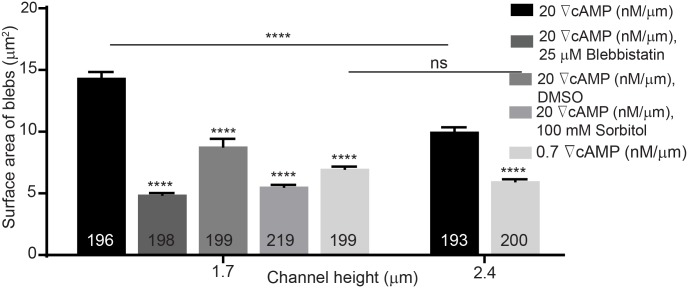
Increasing the cAMP gradient from 0.7 nM/μm to 20 nM/μm induces the cells to produce blebs with larger surface area. Sorbitol and blebbistatin reduced the bleb surface area. Bleb numbers are shown on bars. Error bars represent SEM. ****P ≤ 0.0001.

**Fig 12:**
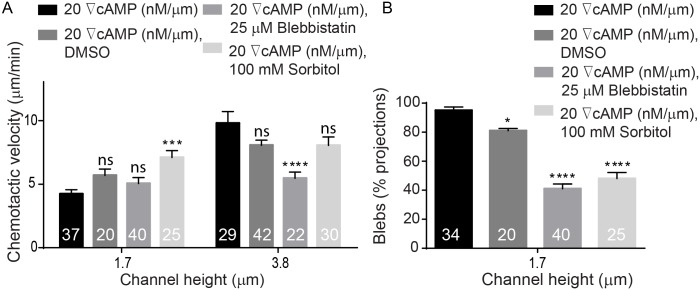
Effects of high osmolarity and blebbistatin on the chemotactic velocity of cells and on the percentage of blebs utilized by the cells. **(A)** High osmolarity buffer led to an increase in cell velocity at 1.7 μm and did not impact the velocity of the cells at 3.8 μm. Additionally, blebbistatin did not impact chemotactic velocity at 1.7 μm and reduced cell velocity at 3.8 μm. **(B)** Cells utilized higher percentage of pseudopods in high osmolarity buffer and after treatment with blebbistatin in 1.7 μm-tall channels. Cell numbers are shown on bars. Error bars represent SEM. *P ≤ 0.1, ***P ≤ 0.001, ****P ≤ 0.0001.

We have shown that *Dictyostelium* cells in confined microfluidic channels move by using blebs, consistent with earlier observations under agarose and our experiments suggest that microfluidic confinement and steep cAMP gradients shift the balance between actin polymerization and myosin contractility, leading to increased intracellular pressure and bleb formation. Because myosin II activity in *Dictyostelium* is stimulated by cAMP and regulated by phosphorylation through downstream guanylyl-cyclases and cyclic-GMP-binding proteins [52,53], we suggest that cortical accumulation of myosin II mediated by confinement leads to increased cortex contractility potential. This mechanical-chemical coupling is supported by previous work in zebrafish progenitor cells, which can be promoted to bleb by increasing myosin II activity through biochemical stimuli [28] and also our experimental observations of how bleb surface area is influenced by confinement height and gradient steepness (Fig 11). At high cAMP steepness (20 nM/μm), for example, the average bleb surface area increases from 10 μm^2^ at a channel height of 2.4 μm to 15 μm^2^ when cells are confined at 1.7 μm. Additionally, when cells were treated with 25 μM blebbistatin, an inhibitor of myosin II, they used less and smaller blebs compare to untreated cells at 1.7 μm. The chemotactic velocity of treated cells, however, remained uninfluenced. We speculate that the increased percentage of pseudopodia can promote higher cell velocity, but the inhibition of myosin II then reduces this velocity. Therefore, these two affects counteract each other and the chemotactic velocity remains largely the same. In cells migrating in channels 3.8 μm in height where they form few blebs, blebbistatin treatment led to a reduction in the chemotactic velocity. We also investigated the behavior of the myo-II-null cells, Myo II: GFP- PDM 181. These cells were not able to invade the 1.7 μm tall channels. However, they migrated across 2.4 μm and 3.8 μm without using blebs and only using only pseudopods. Moreover, when compared to a control experiment, Myo II: GFP- myo II PDM 181 cells were able to invade into all three gradient channels and used 36% blebs during migration at 2.4 μm channels and no blebs at 3.8 μm. Lastly, when the internal cell pressure was reduced through the use of a high osmolarity buffer, cells migrated with less blebs. *Dictyostelium* cells were coerced to migrate across a high (20 nM/μm) gradient in DB buffer containing 100 mM sorbitol, which reduces the osmotic pressure drop across the membrane. We found that cells confined in 3.8 μm channels were not influenced by sorbitol. However, cells confined in 1.7 μm channels formed smaller blebs (Fig 11), migrated with a faster pseudopodia-driven velocity (Fig 12a) and produced significantly fewer blebs (Fig 12b).

Based on our results, we therefore suggest that when stimulated in a steep cAMP gradient under confinement, cells are capable of contracting the cortex to a greater extent than when non-confined due to the increased local availability of myosin II. This contraction leads to increased internal cell pressure and a greater rate of bleb formation during migration. When this internal pressure is reduced with the addition of sorbitol, blebbistatin, or through the use of Myo II-null cells, the rate of formation and the size of these blebs are reduced.

## Supporting Information

S1 Fig Illustration of the experimental set up. (**A**) Constant pressure system for delivering development buffer and cAMP solutions to the microfluidic device. (**B**) Depiction of microfluidic gradient generator device used to study chemotactic *Dictyostelium* cells migrating under confinement. A linear gradient produces constant relative cAMP concentration over the entire channel length while the local cAMP concentration is low at the inlet of the gradient channel and approaches the concentration of the cAMP solution used to form the gradient at the outlet.
(TIF)

S2 Fig Bleb motility is not regulated by the absolute concentration of cAMP. (**A**) The percentage of blebs utilized *Dictyostelium* cells in the vicinity of the inlet and outlet of the gradient channels remained constant. The first bar in each group represents the percentage of blebs at low cAMP concentration and the second bar corresponds to the high concentration end of the gradient. Cell numbers are shown on bars. Error bars represent SEM.
(TIF)
