# Supplementary figures and images for: Cell Blebbing in Confined Microfluidic Environments
Source: PLoS One. 2016 Oct 5;11(10):e0163866. doi: 10.1371/journal.pone.0163866 (PMC5051935; doi:10.1371/journal.pone.0163866)

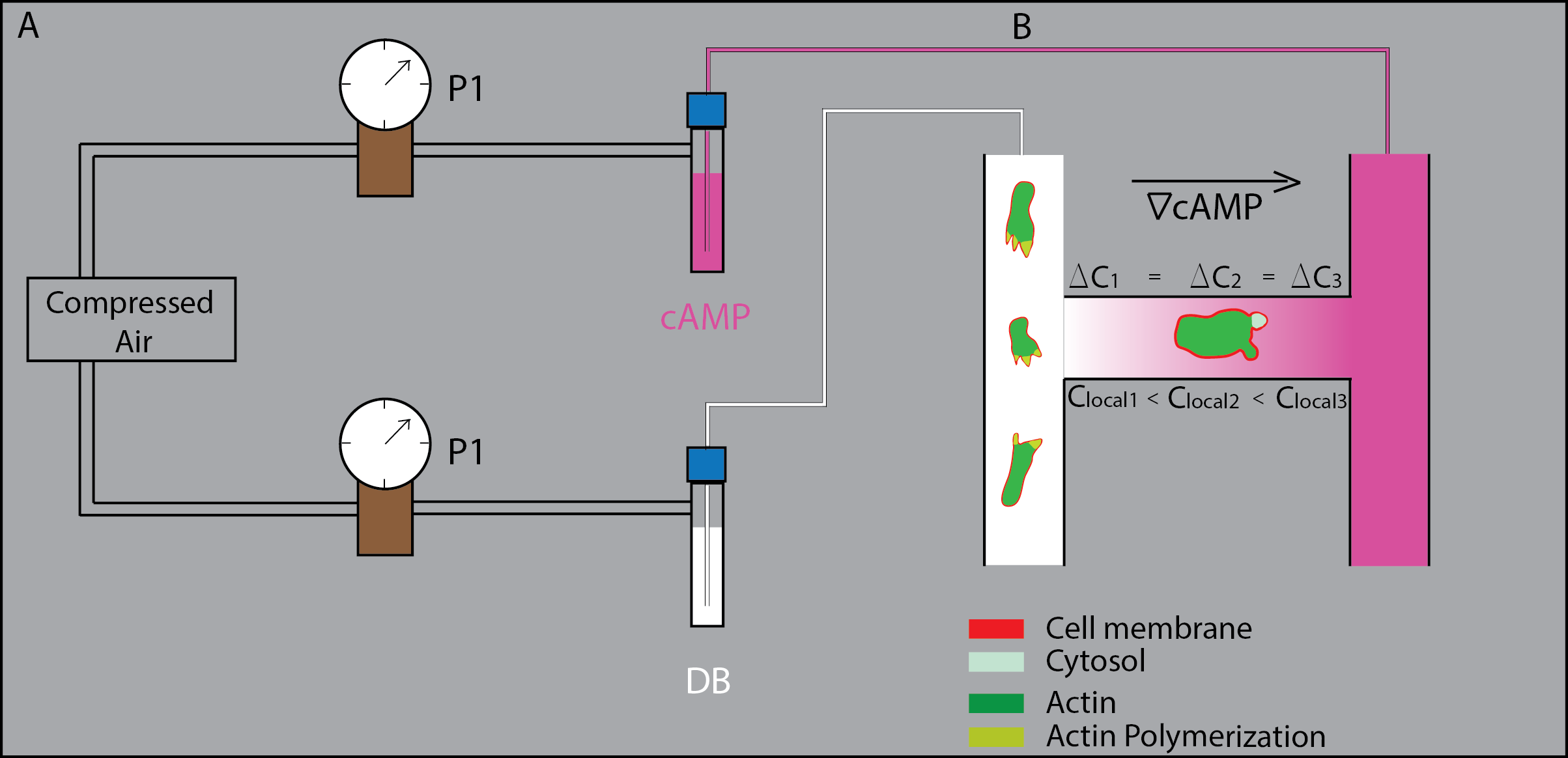

Supplement: S1 Fig — (A) Constant pressure system for delivering development buffer and cAMP solutions to the microfluidic device. (B) Depiction of microfluidic gradient generator device used to study chemotactic Dictyostelium cells migrating under confinement. A linear gradient produces constant relative cAMP concentration over the entire channel length while the local cAMP concentration is low at the inlet of the gradient channel and approaches the concentration of the cAMP solution used to form the gradient at the outlet. (TIF) [file pone.0163866.s001.tif]

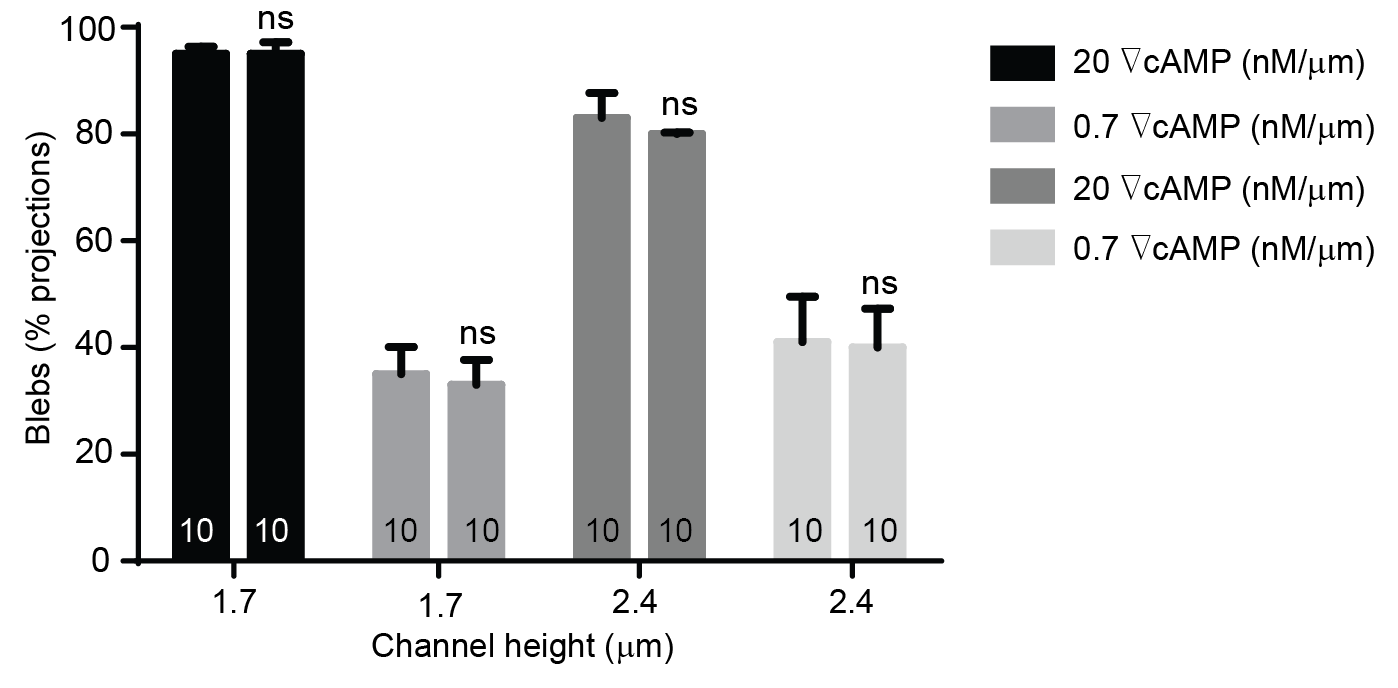

Supplement: S2 Fig — (A) The percentage of blebs utilized Dictyostelium cells in the vicinity of the inlet and outlet of the gradient channels remained constant. The first bar in each group represents the percentage of blebs at low cAMP concentration and the second bar corresponds to the high concentration end of the gradient. Cell numbers are shown on bars. Error bars represent SEM. (TIF) [file pone.0163866.s002.tif]
